# Subclavian thrombosis in a patient with advanced lung cancer: a case report

**DOI:** 10.1186/1752-1947-5-173

**Published:** 2011-05-06

**Authors:** Paul Zarogoulidis, Eirini Terzi, Georgios Kouliatsis, Vasilis Zervas, Theodoros Kontakiotis, Alexandros Mitrakas, Kostas Zarogoulidis

**Affiliations:** 1 University Pulmonary Department, Oncology Unit, "G Papanikolaou" Hospital, Thessaloniki, Greece; 2 University Pulmonary Department, Bronchoscopic Unit, "G Papanikolaou" Hospital, Thessaloniki, Greece

## Abstract

**Introduction:**

Lung cancer is now considered the most common cause of death among cancer patients. Although target biological regimens have emerged in recent years for non-small cell lung carcinoma, the survival and quality of life of patients with this condition still remain low. The five-year survival rate for all stages of lung cancer is 17% or less.

**Case presentation:**

We describe the case of a 53-year-old Caucasian woman who was diagnosed with advanced stage IIIa (T2aN_2_M_0_) non-small cell lung carcinoma (adenocarcinoma) and underwent a complete left upper lobectomy three years ago. After two and a half years of follow-up, she suddenly presented with facial edema and venous distension and was immediately treated for superior vena cava syndrome. Because of a diagnostic check, a major clot was detected in the right subclavian vein. Our patient was informed about treatment options, and she was taken to the catheterization laboratory for percutaneous stenting of the superior vena cava to restore superior vena cava patency.

**Conclusion:**

Lung cancer has a vast number of complications. Superior vena cava syndrome and thrombosis should be considered upon the presentation of a patient with obstructive symptoms. In this case report, even though we expected the clot to be on the side of the former lesion, it was present on the opposite side. Treatment should also start immediately in these patients with clinical suspicion of thrombosis to avoid further complications, even in cases with a differential diagnosis problem. Finally, although patients with non-small cell lung carcinoma have a high incidence of thromboembolic events, anticoagulant treatment is given only as maintenance therapy after a first event occurs.

## Introduction

Lung cancer is one of the leading causes of death in the European Union, with an incidence of approximately 180,000 cases per year [1]. Superior vena cava syndrome (SVCS) is a well-known manifestation of benign and malignant tumors of the upper mediastinum, that causes obstruction of blood flow through the superior vena cava (SVC) [2] in approximately 1.7% to 4% of patients with lung cancer [2,3]. Most of the cases are caused by compression of the SVC by tumors; pure intravascular thrombosis is extremely uncommon and only 0.04% of hospitalized adults have been diagnosed with cancer-related SVC thrombosis [3,4]. Percutaneous treatment via stenting is an accepted strategy as a palliative approach for patients with SVCS if it is impossible to treat the underlying disease, most commonly a metastatic tumor, and when the patient is highly symptomatic [5]. This report discusses a rare case of SVCS by cancer-related thrombosis treated with endovascular stenting, resulting in complete restoration of blood flow and immediate relief of symptoms without any complications.

## Case presentation

**Figure 1:**
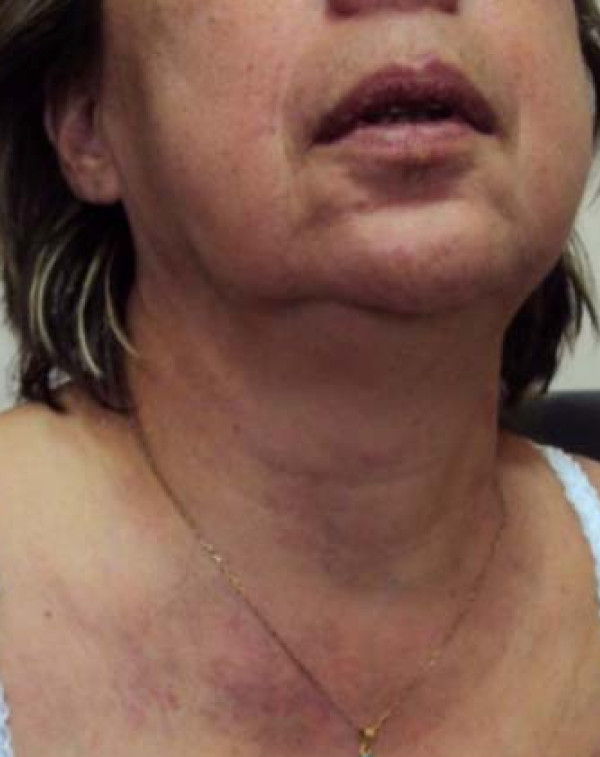
**Image showing facial edema and venous distension**.

**Figure 2:**
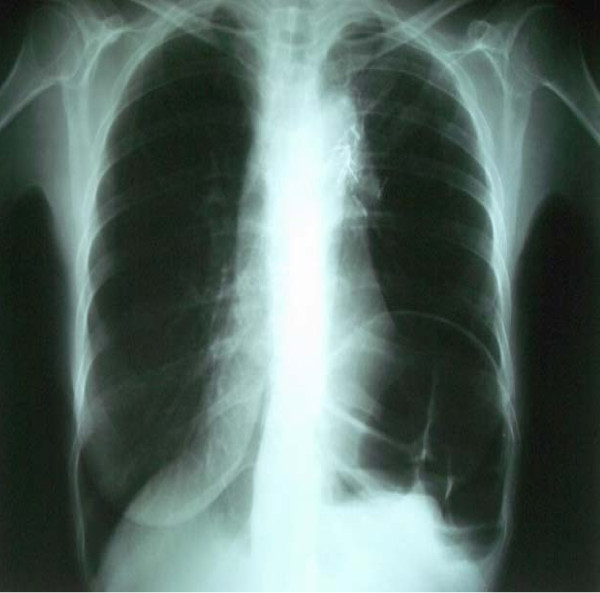
**Chest radiograph taken on the day of the thrombus diagnosis**.

A 53-year-old Caucasian woman consulted our department complaining of progressively worsening facial swelling and a feeling of "tension in the head," which she had first experienced eight days previously and had gradually worsened. Our patient had a history of locally advanced lung cancer (stage T2aN_2_M_0_-IIIa). It was first diagnosed three years before as a left upper lobe mass attached to the mediastinum and was treated with left upper lobe complete resection. The pathologic examination revealed poorly differentiated adenocarcinoma. Our patient was subsequently treated with six cycles of taxane and platinum chemotherapy and radiotherapy at the primary site. It was decided to initiate a complete chemotherapy regimen for locally advanced lymph node disease N_2_. After two and a half years of follow-up, our patient was diagnosed with progressive disease (left supraclavicular nodes and sternum bone metastases), and at the time of examination, she was not receiving any treatment. Her physical examination revealed facial edema and thoracic and upper limb venous distension (Figure 1). The differential diagnosis included central venous obstruction or thrombosis, including SVCS. A chest radiograph showed no progression of the disease in either hemithorax at the time of symptom presentation (Figure 2). Her blood examination results were as follows: white blood cell count 5770/mm^3^, hemoglobin 8.4 g/dL, platelets 253 × 10^4^/mm^3^, glucose 92 mg/dL, creatine 1.23 mg/dL, aspartate aminotransferase 20IU/L, alanine aminotransferase, 10IU/L, alkaline phosphatase 107IU/L, lactate dehydrogenase 382IU/L, albumin 2.8 g/dL, total bilirubin 0.6 mg/dL, sodium (Na^+^) 141.4 mEq/L, potassium (K^+^) 4.3 mEq/L, calcium (Ca^2+^) 8.9 mg/dL, uric acid 4.1 mg/dL, international normalized ratio (INR) 0.94, and D-dimers 4300 μg/mL.

**Figure 3:**
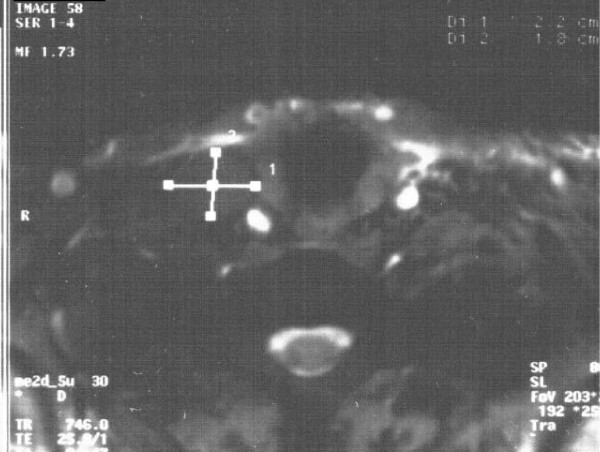
**Contrast-enhanced CT of the chest demonstrating thrombosis at the level of the right subclavicular vein**.

**Figure 4:**
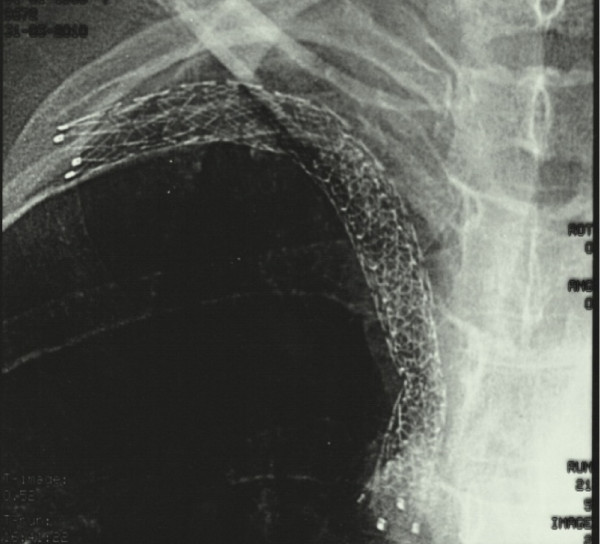
**Chest radiograph showing the stent placed in the right subclavicular vein and superior vena cava through thrombosis**.

Our patient was clinically diagnosed with SVCS, and contrast-enhanced computed tomography (CT) was performed to confirm the diagnosis. Enhanced neck CT demonstrated a major thrombus-like lesion inside her right jugular vein (Figure 3). The standard therapeutic treatment modality for SVCS is radiotherapy, but because of the CT angiography findings, our patient was sent to the catheterization laboratory for percutaneous stenting. The stenosis in the right jugular vein was transversed with a 0.35 inch guidewire (Bioart, Tokyo, Japan) and an 8Fr guiding catheter (Boston Scientific, Natick, MA, USA). The obstruction was dilated using a 3.0 mm×80 mm balloon, and a stent (Dynamic Balloon-Expandable Stent; Abbott Laboratories, Abbott Park, IL, USA) of equal size (3.0 mm×56 mm) was implanted in her right subclavical vein. After the stent placement (Figure 4), our patient showed immediate relief of her symptoms, and she was discharged home the day after the procedure on anticoagulant therapy (warfarin, to maintain prothrombin time INR between 2.0 and 2.5). Five months after the stenting procedure our patient is still asymptomatic with no signs of SVCS on physical examination, and she is on oral anticoagulation treatment with an optimal therapeutic INR level.

## Discussion

Malignancy in non-small cell lung carcinoma (NSCLC) is the most common cause of SVCS, as a result of either compression of the SVC by an adjacent tumor or compression by mediastinal lymph nodes. However, because the velocity of blood flow in the SVC is too fast to permit blood thrombosis, the development of SVC thrombosis alone is extremely rare [4-6]. In patients with neoplastic disease, a syndrome can occur with recurrent thrombosis in unusual areas (including SVC), known as Trousseau's syndrome. Reported varieties of underlying malignancies in patients with Trousseau's syndrome include pancreatic cancers (32.5%), lung cancers (23.6%), gastrointestinal cancers (17.1%) and other cancers (26.8%) [7]. The main pathophysiologic mechanisms of Trousseau's syndrome are malignancy-related hypercoagulability and tumor cell injury of the vascular endothelium, followed by platelet aggregation and activation and consumption of anti-thrombin III and thrombomodulin. Takeda *et al. *[6] reported the case of a patient with SVC thrombosis in which the major etiologic pathway was suggested to be metastasis of cancer cells to the SVC vessel endothelium from lymphatic drainage through the thoracic duct leading to the left innominate vein via the left jugulosubclavicular angle. The attachment of metastatic cells to the vessel endothelium was considered as the trigger to thrombus formation, considering the existence of malignant cells in the intra-SVC thrombus.

SVCS is often diagnosed clinically on the basis of symptoms of venous congestion, including facial and neck swelling, dyspnea and headache. Venous Doppler ultrasonography, contrast-enhanced CT and magnetic resonance imaging are contributory diagnostic modalities when the diagnosis is unclear [8]. In malignancy-associated SVCS, treatment is generally directed at the malignant disease process. Treatment modalities available for SVCS include local radiation (radiation therapy to the malignant process to provide decompression), chemotherapy, steroids (useful only for patients with SVC obstruction as a result of lymphoma) and occasionally diuretic therapy [2].

Endovascular options for the treatment of patients with SVCS in the setting of lung cancer include thrombolysis, angioplasty and stent placement. The use of angioplasty and stenting has developed over the past 15 years. Initially, SVC stents were used in patients who failed to respond to traditional therapy or whose symptoms recurred after traditional therapy. In this patient population, SVC stents have had dramatic technical and clinical results; relief of SVC obstruction has been demonstrated in more than 90% of these patients and obtained with a delay of 24 to 72 hours [5,9]. The researchers in all of these studies investigated the efficacy of stenting in SVC obstruction in the setting of both small cell lung cancer and NSCLC, but none have reported results individually by histological type. Given the excellent results in this patient population, more recently a few authors have suggested that stenting should be used as initial therapy in all patients with malignant SVCS and not only after treatment failure or symptom recurrence after classical treatment. The findings from a large number of case series demonstrate excellent clinical results and low complication rates [10]. With the high success rate of stenting (decreased time to SVC obstruction relapse, increased overall survival and nearly complete and immediate relief of symptoms), endovascular treatment has become the primary safe, consistent, and cost-effective treatment choice for patients with SVCS [5,10]. For stent placement, the patient's condition must be stable enough for the patient to undergo a one to three hour procedure, and coagulopathies should be corrected.

Complications of stent placement have been reported in 3% to 7% of patients with SVCS [11]. The most common complications of this therapy are stent thrombosis and stent migration or misplacement [11]. The risk of stent thrombosis is significantly reduced when long-term anticoagulation with warfarin is used after endovascular stenting [11]. The role of anticoagulation has been debated in the literature. Anticoagulation therapy is often prescribed for patients with SVC obstruction or after stenting, although its effectiveness has never been demonstrated, and the type (heparin, warfarin, aspirin or ticlopidine) and length of preventive treatment remain controversial [5,10,11]. Some authors recommend that all patients with new stents undergo short-term (three to six months) anticoagulation while endothelialization takes place, because significant pulmonary emboli may result. Others recommend long-term anticoagulation in this setting, and others suggest that anticoagulation must be used with caution in patients with malignancies [12]. Other complications reported in the literature include infection, pulmonary embolus, hematoma at the insertion site, bleeding, thoracic pain during balloon inflation [5,9], perforation or rupture of the vein, cardiac tamponade, acute cardiogenic pulmonary edema and transient hemidiaphragm elevation [5,9,13,14].

Cancer patients undergoing surgery or bedridden with acute medical illness should receive routine thromboprophylaxis (that is, what is customarily used on the basis of the type of surgery or for patients with acute medical illness). In cancer patients with indwelling central venous catheters, the American College of Chest Physicians (ACCP) advises against using prophylactic doses of low-molecular-weight heparin or mini-dose warfarin (that is, 1 mg/day) for the prevention of catheter-related thrombosis. The routine use of thromboprophylaxis for primary prevention of venous thromboembolic event (VTE) is not recommended for cancer patients receiving chemotherapy or hormonal therapy. The routine use of primary thromboprophylaxis for improvement of survival in cancer patients is also not recommended [15].

In our report, we present the case of a patient with upper left lobe lung disease and cancer-related thrombosis of the right subclavicular vein that led to SVCS after surgical resection. We report this case because we would usually expect the thrombus to form on the left hemithorax because of the regional effects of the cancer cells. Also, at the time of symptom presentation, our patient did not have lung disease. This case report illustrates the effectiveness of vascular stenting in the management of SVCS in a lung cancer patient with subclavicular thrombosis. Because SVC obstruction is a highly stressful complication for patients with lung cancer, we used endovascular stenting as the main therapeutic intervention for an effective and fast-acting procedure. Our patient was in addition receiving anticoagulation therapy for the prevention of further thrombosis and recurrence. We believe that, given the efficacy of endovascular stenting, future patients will undergo vascular stenting as the first-line treatment despite the elevated cost of this relatively new technique.

## Conclusion

Lung cancer is a well-known predisposing factor for thrombosis. Central venous thrombosis should be included in the differential diagnosis of a patient with symptoms that could be attributed to venous obstruction. The results achieved with endovascular stents in the treatment of SVCS of malignant causes are excellent, and percutaneous endovascular stent insertion is an effective treatment for palliation of SVCS because it provides immediate and sustained symptomatic relief. The high response rates, quickness of effect and safety make this palliative treatment a useful tool and a candidate for being the potential standard in the management of SVC obstruction. It has not yet been established whether cancer patients without locally recurrent disease should receive anticoagulant therapy. The risk of deep venous thrombosis is low in cancer patients without additional risk factors. This fact is in accordance with the ACCP guidelines, which do not recommend routine prophylaxis for VTE prevention in cancer patients in itself [15]. The risk steadily increases with the number of risk factors. Thus, risk assessment tools seem to be sensible to stratify prophylactic regimens in these patients. Risk assessment is mandatory to identify patients at high risk with respect to the application of prophylactic therapeutic regimens, which have to be carefully investigated in randomized clinical studies.

## Consent

Written informed consent was obtained from the patient for publication of this case report and any accompanying images. A copy of the written consent is available for review by the Editor-in-Chief of this journal.

## Competing interests

The authors declare that they have no competing interests.

## Authors' contributions

PZ was responsible for the medical care of the patient and was a contributor in writing the manuscript. ET was a major contributor in writing the manuscript. GK was also responsible for the patient's medical care. VZ was the vascular surgeon responsible for placing the stent. TK diagnosed the patient on the basis of bronchoscopy. AM was the surgeon who performed the lobotomy. KZ is the head of the department and responsible for the patient's medical care. All authors read and approved the final manuscript.
